# Interventional varicose vein therapy using endoluminal laser ablation (1940 nm) in patients with Ehlers‐Danlos syndromes: two case reports

**DOI:** 10.1111/ddg.15812

**Published:** 2025-09-19

**Authors:** Dennis Braß, Iliana Tantcheva‐Poór, Markus Stücker

**Affiliations:** ^1^ Department of Dermatology Venereology and Allergology Center for Vein Treatment at the Section of Dermatology and Vascular Surgery Catholic Hospital Bochum gGmbH, Bochum; ^2^ Department of Dermatology and Venereology University of Cologne

**Keywords:** chronic venous insufficiency, endoluminal ablation, Ehlers‐Danlos syndrome, Varicose vein

## Abstract

In patients with Ehlers‐Danlos syndrome, the presence of chronic venous insufficiency poses a particular challenge for physicians when determining the indication for surgical treatment. Due to vascular fragility, complications such as vascular tears or ruptures with difficult hemostasis and potentially life‐threatening consequences occur particularly during open surgical procedures. The degree of vascular vulnerability varies among the different subtypes of Ehlers‐Danlos syndrome but can be observed across all subtypes. We performed endoluminal laser ablation close to the saphenofemoral junction of insufficient truncal veins in two patients with Ehlers‐Danlos syndrome using a 1940‐nm radial fiber and achieved adequate vessel closure without intra‐ or postoperative complications.

This procedure, described here for the first time, represents an approach between conservative varicose vein therapy and open surgical treatment and may be considered in patients with Ehlers‐Danlos syndrome, particularly in cases of insufficient symptom control or a complicated disease course.

## INTRODUCTION

Ehlers‐Danlos syndromes are a heterogeneous group of hereditary connective tissue diseases with currently thirteen subtypes.^1^ These syndromes are rare; the incidence of the most common monogenetic subtype – classic Ehlers‐Danlos‐Syndrome (cEDS) – is estimated at 1:20,000.^2^ Mutations resulting in this syndrome have been found on 20 different genes and can follow either autosomal dominant or autosomal recessive heredity. Depending on the individual mutation, the genetic alterations will mainly cause defects in the primary structure of fibrillary collagens I, III, and V. Additional dysfunction of collagen‐processing enzymes or collagen‐associated extracellular matrix (ECM) proteins such as tenascin X, proteoglycans, or participating transcription factors may also occur.^1^, ^2^

All Ehlers‐Danlos syndromes are characterized by joint hypermobility, skin hyperelasticity, and tissue fragility in various organs (skin, blood vessels, hollow organs, eyes); however, manifestations differ within and between the subtypes.^2^ While epidemiological data are currently still lacking^3^, there are reports of an increased prevalence of chronic venous insufficiency in patients with Ehlers‐Danlos syndrome.^4^ This mainly affects patients with vascular Ehlers‐Danlos syndrome (vEDS),^3^, ^5^ but venous complications have also been reported in patients with cEDS, spondylodysplastic (spEDS), and kyphoscoliotic Ehlers‐Danlos syndrome (kEDS).^4^ Such a situation makes it difficult for phlebologists to arrive at a clear indication in favor of surgical treatments, particularly in view of the increased perioperative risk. There are currently no specific recommendations, either at a national or international level.^2^ Apart from the classic, open surgical procedures, endoluminal laser ablation offers a minimally invasive option for the treatment of truncal varices. We will present two case reports below.

## CASE 1

A 42‐year‐old man with *SLC39A13*‐associated spEDS first presented at our clinic in 2023 because of stage C4a chronic venous insufficiency. The patient reported that he had had bilateral inguinal crossectomy due to insufficiency of both great saphenous veins in the area of the saphenofemoral junctions in 1996. Surgical crossectomy was again performed bilaterally in 2009 due to clinical relapse. No complications were reported in association with these interventions. Repeat side branch stripping was initiated in 2018 but had to be terminated prematurely due to pronounced vessel fragility and severe bleeding. The patient presented at our clinic to explore possible therapeutic options for his bilateral large‐lumen side branch varicosis in the thighs and calves as well as accompanying trophic skin alterations on the distal calves (*purpura jaune d'ocre*). Duplex‐ and Doppler‐supported ultrasound of both legs was performed for diagnosis. This revealed truncal vein insufficiency of the small saphenous vein on the right leg (Hach stage II, reflux duration 2.5 s, maximum diameter 3 cm distally from the junction: 9.9 mm), incomplete truncal vein insufficiency of the small saphenous vein in the left leg with short‐distance aneurysmal dilation (reflux duration > 2 s, maximum diameter 9.5 mm), and finally bilateral large‐lumen side branch varicosis in the thighs and calves. Further inguinal junction relapses were not detected. The patient's surgical history showed connective tissue fragility in the context of his known spondylodysplastic Ehlers‐Danlos syndrome, while at the same time he had a medical indication for repairing the truncal vein and side branch varicosis. This led to a comprehensive discussion of out‐patient, endoluminal laser ablation of the small saphenous vein on the right leg, performed as minimally invasive surgery with tumescence anesthesia, utilizing a 1940 nm radial laser but forgoing mini‐phlebectomy, with subsequent sclerotherapy of the accompanying side branch varicosis. The total energy applied during the procedure was 1107.5 Joule (which corresponds to a linear energy dose of 185 J/cm); the radial fiber was slowly and steadily withdrawn via automated retraction over a period of 149.5 s, from the saphenopopliteal transition in a distal direction. The procedure was performed under constant ultrasound control to detect any vascular injuries. Altogether, a 6 cm section of insufficient vein in the small saphenous vein on the right leg was thus treated with laser ablation. There were no complications such as vessel fragility or postoperative bleeding from the puncture site on the right calf.

**FIGURE 1:**
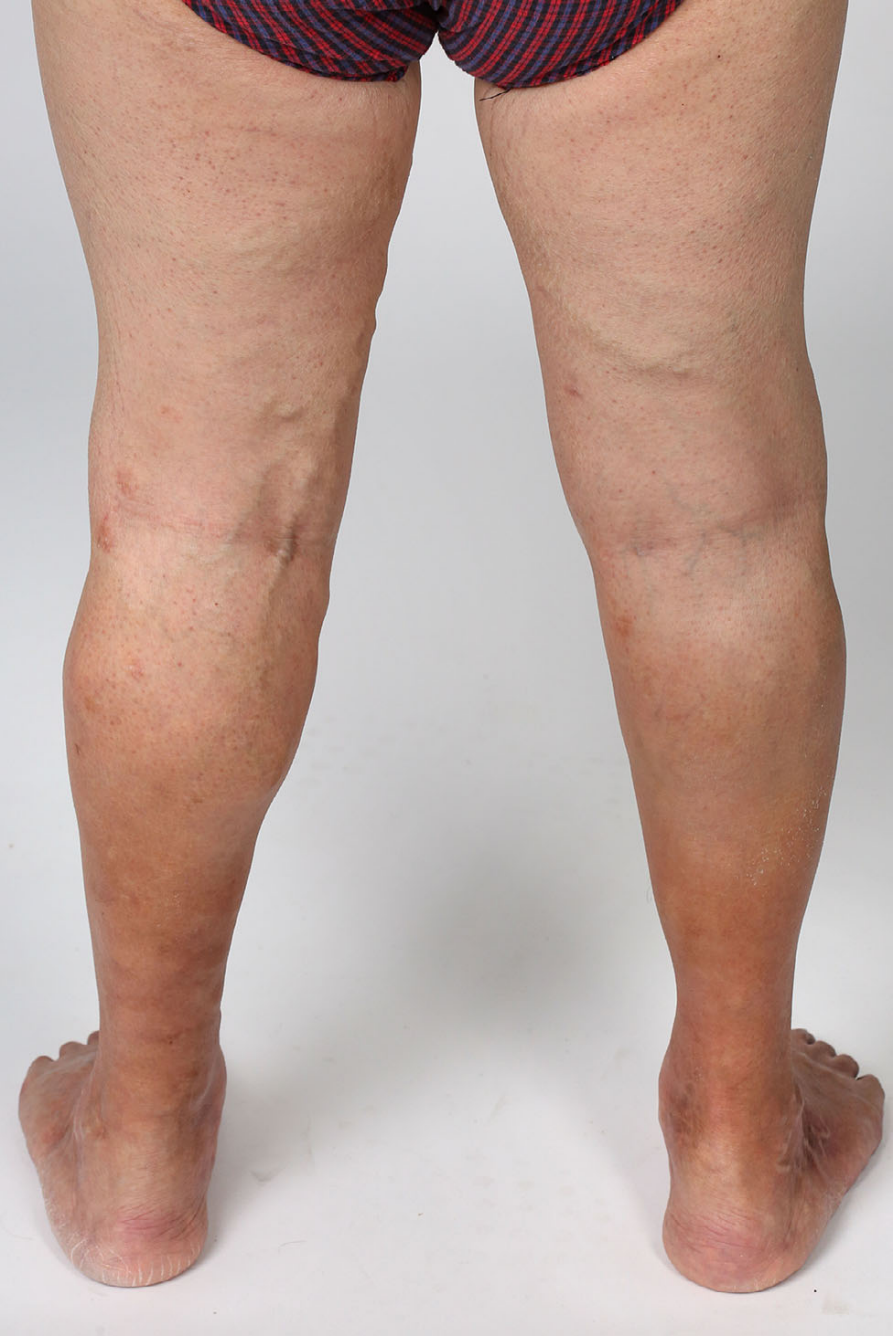
Findings on initial presentation: Pronounced lateral branch varicosis of the dorsomedial thigh and lower leg on both sides with strong *purpura jaune d'ocre*.

**FIGURE 2:**
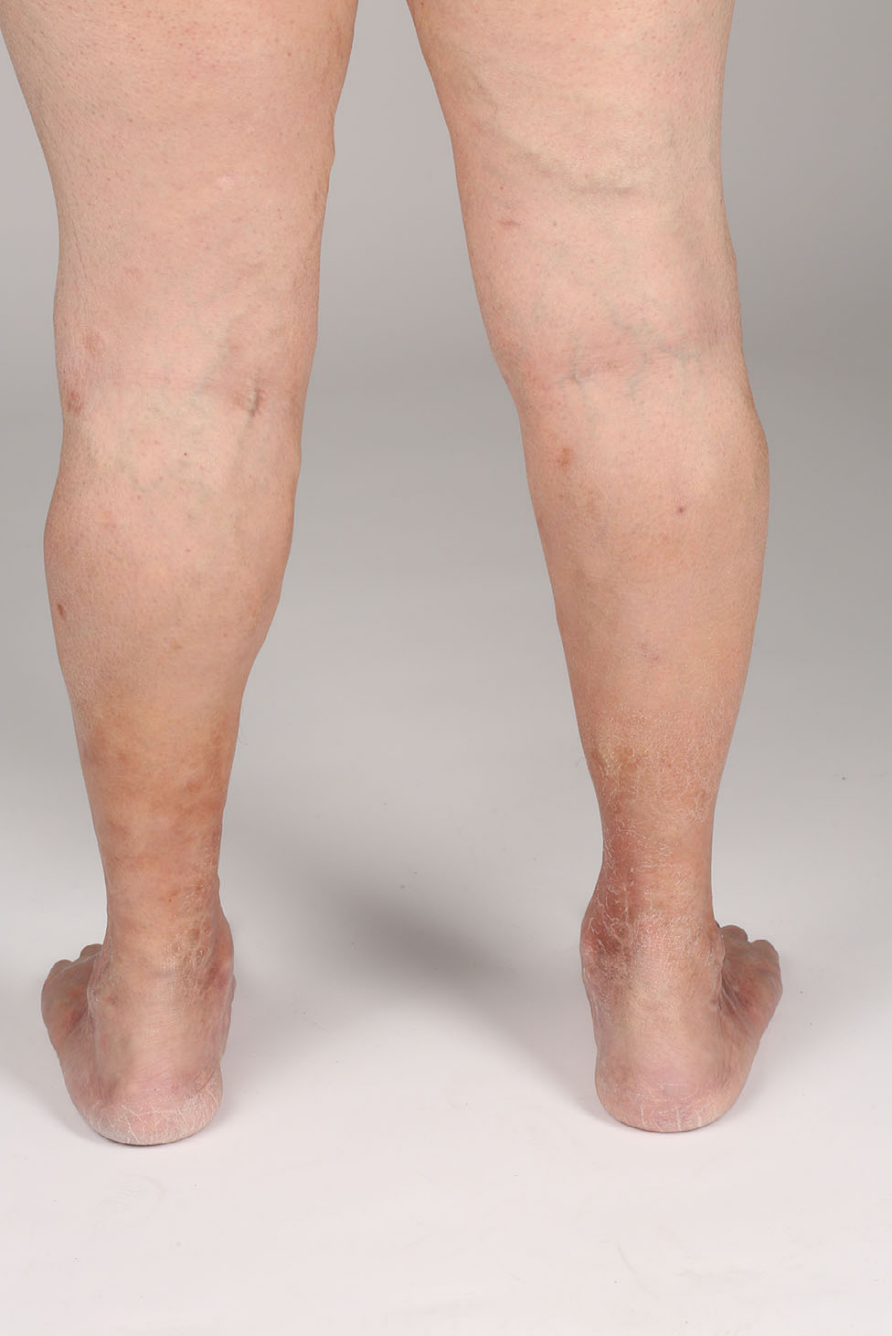
Postoperative findings under ongoing sclerotherapy: Reduction of the side branch varicosis and hyperpigmentation of the thighs and lower legs on both sides.

Postoperative treatment consisted of compression bandaging of the right calf with elastic medium‐stretch bandages (Salva‐Last^®^) until the next day. Monitoring of the results was performed on postoperative days 1 and 8, and Duplex sonography confirmed complete occlusion of the treated small saphenous vein. The side branch varices were treated with foam sclerotherapy (Polidocanol 0.5%, 1 mL Polidocanol with 4 mL air) with 2 mL per treated vessel. This was first performed two weeks after surgery, starting on the thighs and continuing in a distal direction. Foam formation was achieved via a three‐way valve. To date, seven treatment sessions have been completed without any complications, each resulting in satisfactory occlusion of the treated vessels. There were isolated occurrences of painful sclerothrombi which were successfully treated symptomatically via puncture, thrombus expression, heparin ointment dressings, and continuation of compression therapy with a medical class II compression stocking. Duplex sonography one year after the procedure showed continued complete occlusion of the treated small saphenous vein (Figures 1, 2).

## CASE 2

In the summer of 2023, a 64‐year old female patient with known cEDS presented at our clinic. She had by then already had chronic venous insufficiency in the left leg for many years, with truncal vein insufficiency of the great saphenous vein. She also had a history of multiple superficial leg vein thromboses both in the area of the insufficient truncal vein and the area of adjoining side branches. Surgical treatment had not yet been performed. Clinical findings included side branch varices as well as *purpura jaune d'ocre* on the left leg. Duplex sonography revealed complete truncal vein insufficiency of the great saphenous vein with insufficiency of the terminal saphenofemoral valve (Hach stage II–III, maximum diameter 3 cm and 15 cm distally from the saphenofemoral junction: 10.9 mm and 9.5 mm; stage C4a).

**FIGURE 3:**
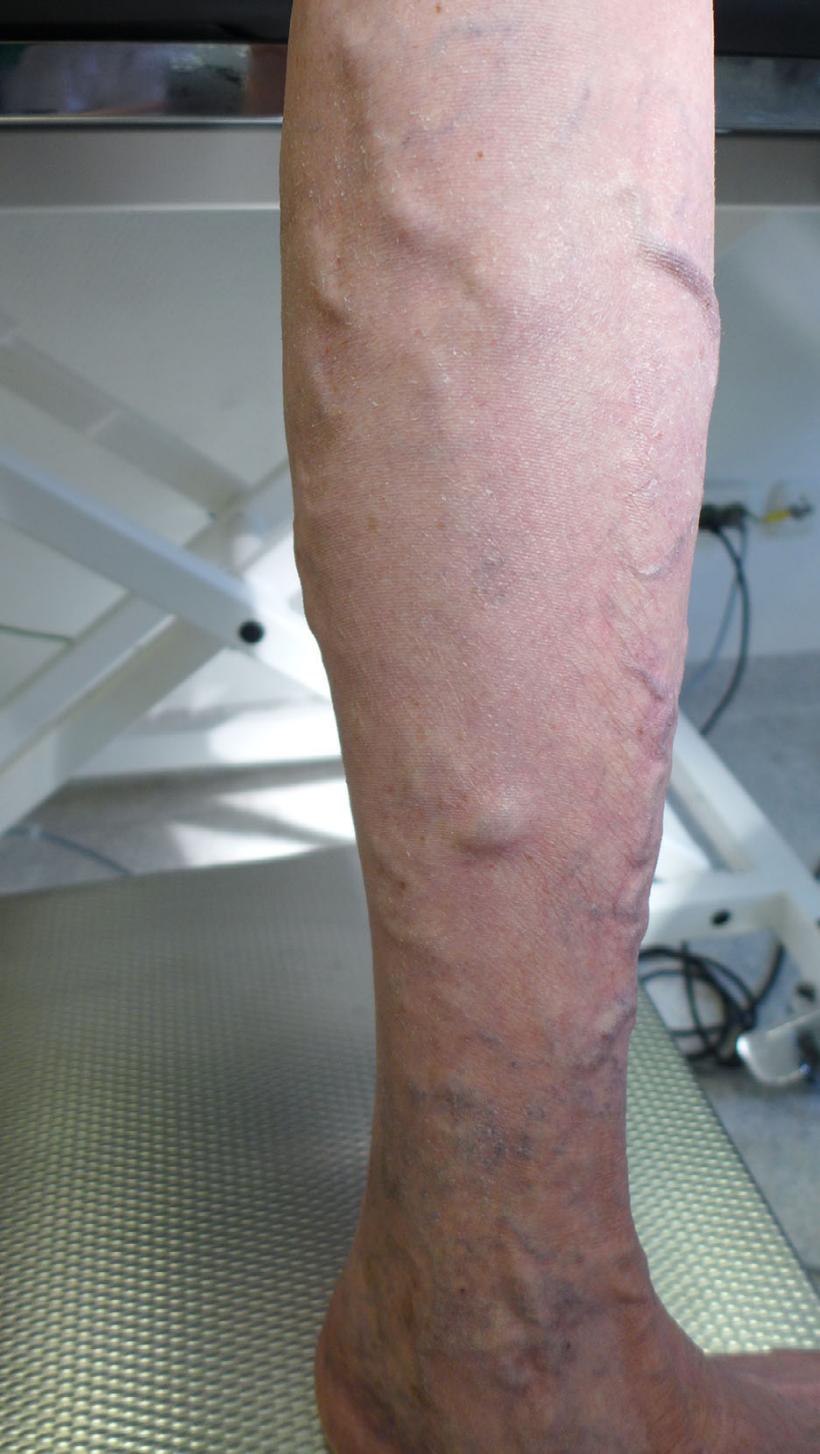
Pre‐therapeutic findings: Large‐lumen lateral branch varicosis of the left lower leg with distal reticular varicosis and *purpura jaune d'ocre*.

**FIGURE 4:**
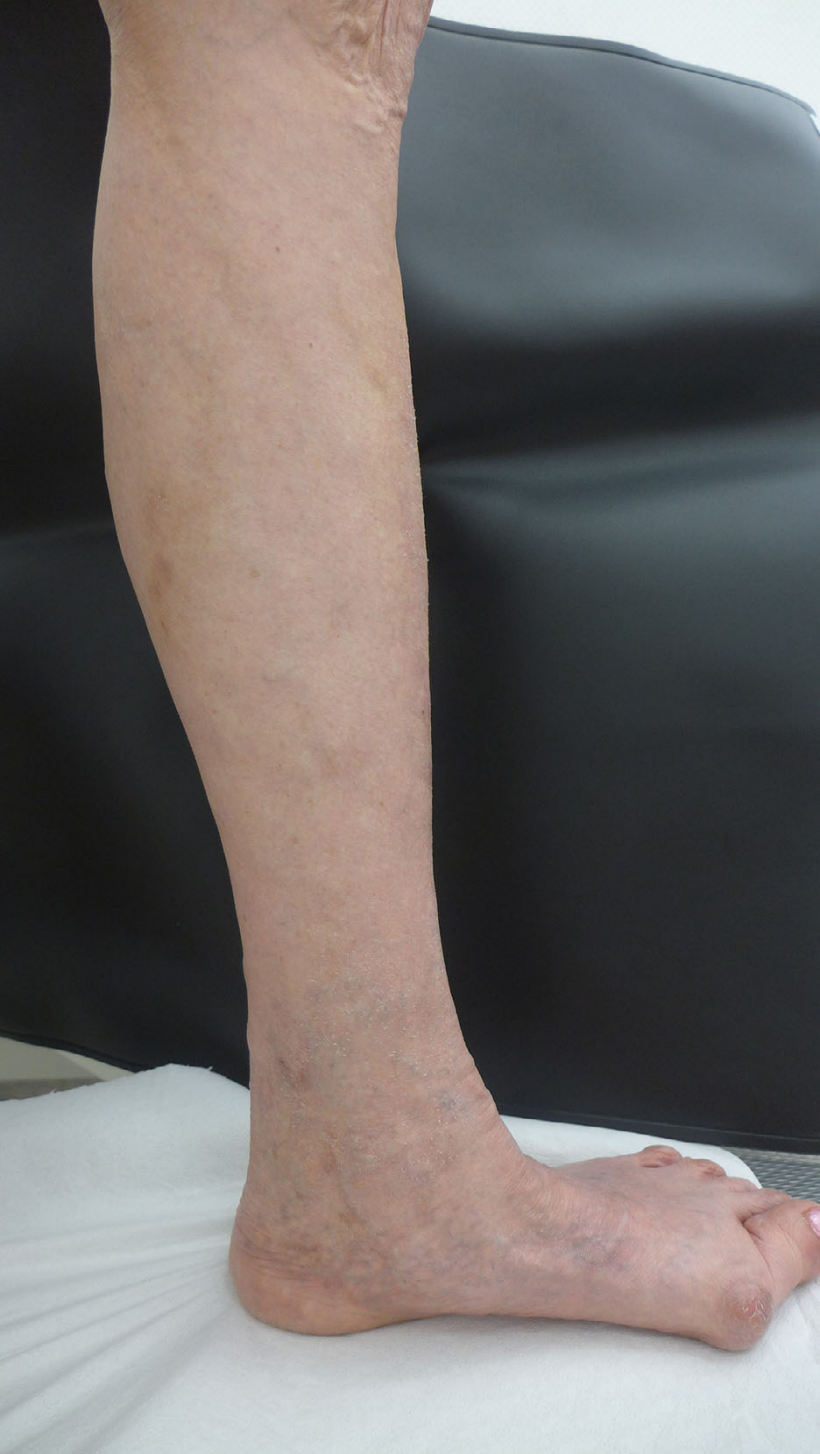
Postoperative findings: Significant reduction of the formerly large‐lumen lateral branch varicosis, the reticular varicosis and the hyperpigmentation of the left lower leg.

Due to progression of her chronic venous insufficiency with acute complications (superficial thromboses) as well as chronic complications (hyperpigmentation), we discussed the medical indication for surgical repair with the patient. Given the context of the known EDS, we decided on endoluminal laser ablation of the great saphenous vein on the left leg, forgoing additional mini‐phlebectomy of the side branch varices. Minimally invasive surgery was performed with tumescence anesthesia, utilizing a 1940 nm radial laser with a total energy application of 3834.3 J and slow retraction of the glass fiber over a total length of 37 cm within a period of 454.6 s. We aimed at a linear energy dose of 160 J/cm for the proximal 5 cm of the great saphenous vein, and 80 J/cm for the more distal sections. There were no intraoperative complications such as vessel injury or bleeding. After surgery, the left thigh and calf were compressed until the next day; both clinical examination and duplex sonography were performed as follow‐up on days 1 and 8 after surgery. The truncal vein treated with endoluminal ablation showed the intended occlusion; extensive hematoma or postoperative pain did not occur. Subsequently, two out‐patient sessions for sclerosing of the side branch varices were performed, using foam sclerotherapy (Polidocanol 0.5%, 1 mL Polidocanol with 4 mL air, foam formation via a three‐way valve). 2 mL were injected per treated vessel; this led to partial excessive sclerosing reactions in this patient. Compression with heparin ointment dressings and self‐massage was continued, which improved the reactions. The final examination one year after laser ablation showed complete occlusion of the great saphenous vein without remnants at the saphenofemoral junction (Figures 3, 4).

## DISCUSSION

In addition to conservative treatment, surgical therapy is a mainstay in the treatment of symptomatic varicosis, especially if patients have progressive symptoms or complications such as (superficial) thromboses or chronic venous ulcers occur.^6^

EDS patients are a group of special concern because deficiencies in connective tissue synthesis foster the formation of varices at a young age ^3^, ^5^ and because treatment, particularly surgery, is associated with a higher risk of complications. There are individual case reports in the literature on complications with surgical or interventional medical procedures in the arterial system,^2^, ^7^ including a markedly increased risk of vessel injury such as tearing, formation of false or true aneurysms, arteriovenous fistulas, or severe and difficult to manage bleeding, in extreme cases resulting in loss of limbs or even death.^3^, ^7^, ^8^

In venous procedures, as well, case reports have shown increased fragility of the vessels, with ruptures associated with minimal manipulation in some cases.^5^, ^8^, ^9^ It is currently unknown if EDS patients have more frequent bleeding requiring transfusion after surgery for varices. In view of these findings, it seems prudent to avoid open surgery especially in vEDS patients,^3^ or, as suggested in earlier research,^5^, ^10^ to restrict surgery to medically unavoidable cases. Interestingly, there are no current suggestions on performing open surgery for insufficient saphenous veins in EDS patients. Older general recommendations and precautionary measures in open surgery^7^ comprise ligature of the saphenous veins with sufficient space between the ligature and the saphenofemoral junction, extreme caution during dissection in the inguinal area due to fragility of vessels and fasciae, use of metal clips in cases of difficult suturing conditions, and leaving skin sutures in place for longer periods.^7^ There are currently only two case reports in the literature covering endoluminal varicosis therapy in patients with vEDS.^3^, ^9^ One case report describes an 18‐year‐old male patient with chronic venous insufficiency who was successfully treated with radio frequency catheter ablation (ClosureFast™  catheter, Covidien) on both great saphenous veins as well as the small saphenous vein on the left leg, and additional sclerotherapy of the side branch varices.^3^ Another article reported on the successful treatment of a 61‐year‐old female patient with a combination of endoluminal laser ablation (ELVeS Radial, Biolitec; LEED 60 J/cm; radial fiber) and transluminal occlusion of the perforator veins (TRLOP).^9^

In two of our patients, endoluminal laser crossectomy of the great and small saphenous veins via radial fiber (1940 nm), combined with subsequent sclerotherapy of side branch varices was performed successfully for the first time. Clinical experience shows that the excessive reactions to sclerotherapy seen in both patients, with transient pain, are comparatively common complications of sclerosing treatment and can be regularly observed in the general population as well. Risks and complications of minimally invasive endoluminal procedures in EDS patients, which need to be taken into account in decision making, are only rarely found described in the current literature. Use of bare‐tipped laser fibers can lead to perforation of the truncal vein walls.^9^ There are also two case reports on the use of short‐wave lasers (810 nm) describing the formation of arteriovenous fistulas.^11^, ^12^ However, this occurred in patients without EDS and may thus be considered a general complication of endoluminal laser ablation with short‐wave laser systems. One case report describes the formation of an arteriovenous fistula after use of a long‐wave (1320 nm) laser system; here as well, this concerned a patient without EDS.^13^ Overall, arteriovenous fistulas are rare complications.^12^, ^13^ There are currently no studies investigating whether EDS patients may be more prone to this type of complication. All in all, use of long‐wave lasers (1320, 1470, 1500, 1920, 1940 nm) and radial emission fibers shows fewer side effects (such as pain, ecchymosis, inflammatory reactions, nerve injury) compared with short‐wave lasers (810–980 nm). Hence, long‐wave lasers should be preferred over short‐wave or bare‐fiber lasers.^6^

The various endoluminal therapeutic procedures are a valid option for EDS patients beyond conservative symptomatic treatments if the latter prove insufficient due to complications or progressive symptoms. Since EDS patients have a genetic predisposition for – sometimes severe – complications during vascular procedures, an individual risk‐benefit evaluation is essential.^2^, ^7^ This is even more relevant in subtypes with major involvement of the vessel walls, such as vEDS.

The cases presented here, as well as the literature, indicate that laser ablation in combination with foam sclerotherapy may offer advantages for periprocedural safety in patients with EDS. There are, however, no long‐term follow‐up data beyond one year. We also do not currently know if the risk of recanalization as well as Duplex and clinical relapses is different in EDS patients as compared to general patients. It is debatable whether, in patients with EDS, possible disadvantages in terms of effectiveness should be accepted in view of the periprocedural safety of endovenous ablation techniques.

### Summary for medical practice

In our center, two EDS patients with truncal varicosis of the great and small saphenous veins received out‐patient endoluminal laser ablation treatment, using a 1940‐nm laser and radial fiber. This treatment was free of complications both during and after the procedure, and the therapeutic results were satisfactory. There are currently no conclusive data on the potential advantages of a 1940‐nm wavelength over the more commonly used 1470‐nm wavelength. As opposed to radio frequency ablation, which requires a 2 cm safety distance to the deep vein, radial fibers allow ablation much closer to ‐ in fact right up to ‐ the junction. It is currently not possible to predict whether this technique may also offer long‐term advantages in terms of effectiveness when compared with radiofrequency ablation. Whereas vessel perforation has been reported in association with bare fibers due to concentrated heat emission, radial emission fibers diffuse thermic energy and thus reduce the risk of perforation. Long‐wave laser systems with radial energy emission are also associated with lower rates of unwanted complications during and after the procedure. In our cases, side branch varices were treated effectively and with a comparatively low risk of complications by sclerotherapy. In our experience, endoluminal laser ablation with a 1940‐nm radial fiber combined with subsequent foam sclerotherapy may offer a safe treatment option for chronic venous insufficiency in EDS patients.

## CONFLICT OF INTEREST STATEMENT

None.
